# Effects of a Dental Gel Over 6 Months on Periodontal Health in Subjects with Stage II and III (Mild and Moderate) Periodontitis

**DOI:** 10.37191/Mapsci-2582-3736-1(3)-019

**Published:** 2019-11-18

**Authors:** Kairong Lin, Thair Takesh, June Hee Lee, Dominique Nhi Duong, Audrey Hoang Nguyen, Ryan Kwan Cheung, Brian L Nguyen, Petra Wilder-Smith, Charles M Cobb

**Affiliations:** 1 Beckman Laser Institute, University of California, Irvine, CA 92612, USA; 2 Department of Periodontics, School of Dentistry,University of Missouri-Kansas City, Kansas City, MO 64108, USA

**Keywords:** Pocket depth, Gingival inflammation, Dentifrice, Dental gel, Periodontal health

## Abstract

**Objective:**

Overall aim of this prospective, randomized, positive controlled, double-blind in vivo study was to identify the effects of a test dental gel containing 2.6% edathamil with an added carrier and permeability enhancer vs. a positive control dentifrice on periodontal health measures in patients with Stage II and III periodontitis.

**Methods:**

In this prospective double-blinded, randomized study, 33 subjects were randomly assigned in a 1:1 ratio to brushing their teeth with either the test gel (LivFresh^®^, Livionex Dental Gel, Los Gatos, CA 95030) or the positive control toothpaste (Crest ProHealth^®^, P&G, Cincinnati, OH 45202).Full-mouth gingival index, modified sulcus bleeding index, and periodontal pocket probing depths were recorded for all teeth at baseline, and on days 90 and 180.Subjects brushed with the study material twice a day.

**Results:**

The test dental gel reduced gingival inflammation and bleeding, as well as periodontal pocket probing depths significantly more than a control dentifrice.

**Conclusions:**

In this pilot study in subjects with Stage II and III periodontitis, a test dental gel was found to improve gingival inflammation and bleeding, as well as periodontal pocket depths significantly better than a control dentifrice.

## Introduction

Persistent oral biofilm is connected with the development of periodontal disease [1,2]. Typically, plaque is removed through oral hygiene measures that include brushing, flossing and other adjunct means of targeted plaque removal.Gingival inflammation typically resolves in response to removal of microbial plaque by means of effective oral hygiene [3]. Inadequate plaque control can cause gingivitis, an inflammation of the gingival tissues that can be reversed by effective plaque control and professional prophylaxis to remove plaque and calculus [4]. It is estimated that up to 50–90% of adults are affected by gingivitis [5]. Persistent gingivitis can cause periodontitis, which is associated with progressively deepening periodontal pockets, and loss of hard and soft tissues that are the supporting structures of the teeth [1,2]. The prevalence of periodontitis in the US population is estimated to approximate almost 50% of adults [6]. Moreover, the adverse systemic health effects of oral biofilm add urgency to the quest for more effective approaches to oral hygiene.

Despite considerable research into improving mechanical plaque control through better toothbrush design, innovative dentifrice formulations and a wide range of adjunctive measures, most individuals struggle to achieve effective and/or adequate plaque removal [4,7–9]. In the United Kingdom a survey found that at least one-third of all teeth had visible plaque in 72% of adults [10].To support a better cleaning action, many dentifrices contain adjuncts formulated to improve cleaning action, discourage plaque reaccumulation, or have an antibacterial effect [5,11,12].

Common ingredients in over-the-counter dentifrices include flavorings, chelators, colors, preservatives, foaming agents, abrasives and detergents.Some recent toothpaste formulations avoid these ingredients as a way of avoiding some of the potential disadvantages of these components. For example, abrasives can produce abrasion of exposed cementum or dentine, especially in older patients with recession and areas of exposed root surface [13].Abrasivenanoparticles have been identified in the bloodstream, raising concern about systemic spread and potential effects at a cellular level in the heart, liver, lungs, and kidneys. There have been reports of potential crossing of the blood-brain barrier by some nanosphere abrasives, providing access to the CNS with unknown risks or effects [13–16]. Side effects of other ingredients in some patients include adverse epithelial mucosal conditions that have been associated withsodium lauryl sulfate and other detergents [13–16] and sensitivity or allergic reactions from artificial coloring agents [16].

One recent approach to improving plaque removal and reaccumulation has been the incorporation of the micro-chelator edathamil into a dental gel (LivFresh®, Livionex Dental Gel, Los Gatos, CA 95030). By binding cations such as iron and calcium, microbial adherence, biofilm formation and bacterial growth are impeded [17]. In order to improve its ability to penetrate into biofilm, a carrier and permeability enhancer are also contained in the dental gel [18]. Several studies have reported that biofilm formation and adhesion are disrupted by this edathamil-containing dental gel, resulting in more effective plaque removal, reduced biofilm reaccumulation, and improved gingival health [19–24].

Overall aim of this prospective, randomized, positive controlled, double-blind clinical in vivo study was to determine identify the effects of a test dental gelcontaining 2.6% edathamil with an added carrier and permeability enhancer *vs.*a positive control dentifrice on periodontal health measures in patients with Stage II and III periodontitis.

## Materials and Methods

### Study Population and Methodology:

This project was performed at the University of California, Irvine, in full compliance with University of California, Irvine IRB-approved protocol #2013–9778. It was registered on Clinicaltrials.gov under the reference number NCT02271815.

#### A. Subjects

### Subjects Inclusion and Exclusion Criteria:

Thirty-three study participants were recruited from students and staff of the University of California, Irvine by IRB-approved flyers and e-mails. The following inclusion and exclusion criteria were applied:

#### 1) Inclusion Criteria

1. Male or female subjects 30 years or older
2. Minimum of 20 teeth
3. Plaque index >2; Gingival Index >1.5; mSBI >1
4. Mild to moderate periodontitis as determined by the Armitage classification (1–4mm CAL)
5. At least 4 siteswith periodontal pocket depth (PPD) ≥4mm
6. Bleeding on probing on >50% of teeth, as determined by single-pass probing depth measurements
7. Willing and able to provide written informed consent
8. Willing and able to comply with study visits as described in the protocol
9. Available for follow up on the telephone.

#### 2) Exclusion Criteria

1. Unable or unwilling to sign the informed consent form
2. Participation in any other clinical study within the last 30 days prior to enrollment into this study
3. Subjects who must receive dental treatment during the study dates
4. History of significant adverse effects following use of oral hygiene products such as toothpastes and mouth rinses. Allergy to personal care/consumer products or their ingredients
5. Presence of any condition, abnormality or situation at baseline that in the opinion of the Principal Investigator may preclude the volunteer’s ability to comply with study requirements, including completion of the study or the quality of the data
6. Within 1 month prior to baseline, any quadrant or maintenance root planning, and/or periodontal surgical therapy
7. Pregnant females
8. Tobacco use
9. Sjögren’s disease
10. Immune deficiency diseases, i.e., HIV or AIDS
11. Poorly controlled diabetes
12. Anti TNF-alpha medication for rheumatoid arthritis
13. Systemic antibiotics in the last 3 months
14. Anti-inflammatory drugs
15. Immune suppressants.

#### B. Design and Protocol

**Figure 1:**
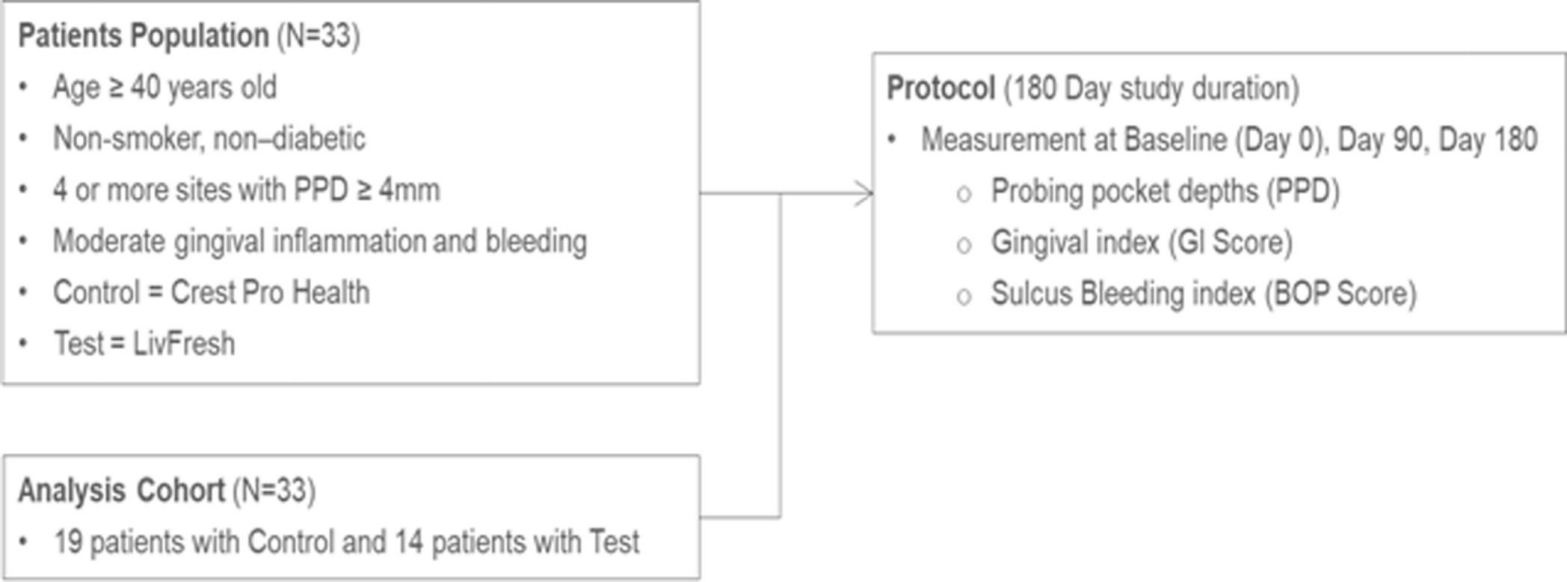
Protocol Summary Flowchart.

After eligibility was determined, and after obtaining informed written consent, subjects were randomly assigned in a 1:1 ratio to treatment with either the test gel or the positive control toothpaste. Full-mouth gingival index, modified sulcus bleeding index, and periodontal pocket depths were recorded for all teeth (TT). Subjects were provided with a new standard Oral B ProFlex® toothbrush (Procter & Gamble, Cincinnati, OH, 45202, USA) and trained using the tell-show-do method in the standard sulcular brushing technique by the same clinician (TT)(Figure 1).

Subjects were instructed to brush with the study material twice a day and to use a pea size amount of the dentifrice provided. They were requested to bring back the empty and partially emptydentifrice tubes at each visit and each tube returned was weighed to measure compliance. Subjects were provided with de-identified plain white, numbered tubes of toothpaste and new toothbrushes at each study visit. They were also asked not to use any other oral hygiene products throughout the study duration.

The first brushing occurred during the Baseline (day 0) visit. Study duration was 180 days; subjects were evaluated at baseline (day 0), day 90, and day 180.

#### C. Products

Test dental gel: Livionex Dental Gel® (Livionex, Los Gatos, CA 95030).

Ingredients: water, sulfonylbismethane, edathamil, stevia, peppermint, menthol essential oils, iota carrageenan gum, konjac gum and lecithin.

Control dental gel: (Crest ProHealth®, P&G, Cincinnati, OH 45202).

Ingredients: Stannous Fluoride, Glycerin, Hydrated Silica, Sodium Hexametaphosphate, Propylene Glycol, PEG-6, Water, Zinc Lactate, Trisodium Phosphate, Flavor, Sodium Lauryl Sulfate, Sodium Gluconate, Carrageenan, Sodium Saccharin, Xanthan Gum, Blue 1.

#### D. Variables Recorded

At each appointment, the following variables were measured by the same blinded, pre-standardized, experienced periodontist (TT):

1. Gingival Index (GI): Löe and Silness Gingival Index [25]
2. Sulcus Bleeding Index (mSBI) [26]
3. Periodontal Pocket Probing Depths (PPD) using a standard UNC probe.

#### E. Data Analysis

The clinical value for each measurement on each tooth on Day 0 was used as baseline value for the subsequent time points of the study. The effects of each dentifrice on each of the variables measured were tested using the % change from baseline for all variables at 3 months and 6 months. A T-test was performed to test for difference between the two treatments. The results are shown in Table 1.

## Results

### A. Subjects

Thirty-three study participants were recruited using IRB-approved flyers and e-mails. Of these 33 recruits, all were enrolled, none were excluded and none dropped out. Thus all 33 study recruits completed the study as instructed.

### B. Study Results

### C. Gingival Index

**Figure 2:**
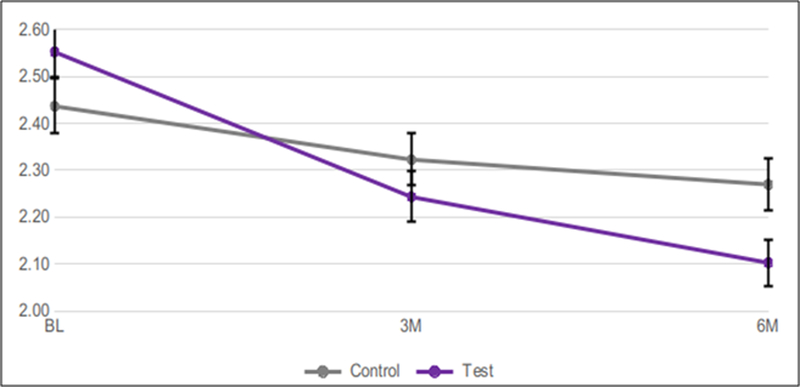
Mean GI over 6 months using test*vs.* control dentifrice. Error bars show standard error of mean (SEM).

Mean Baseline GI measured 2.54 for the test group, and 2.45 for the control group.After 3 months, GI was significantly lower in the test (2.21) *vs.* the control (2.27) group (p=0.0152). This trend was considerably stronger at the 6-month time point, when GI measured 2.04 in the test group*vs.* 2.25 in the control group (p=0.0002)(Table 1 and Figure 2).

### D. Sulcus Bleeding Index

**Figure 3:**
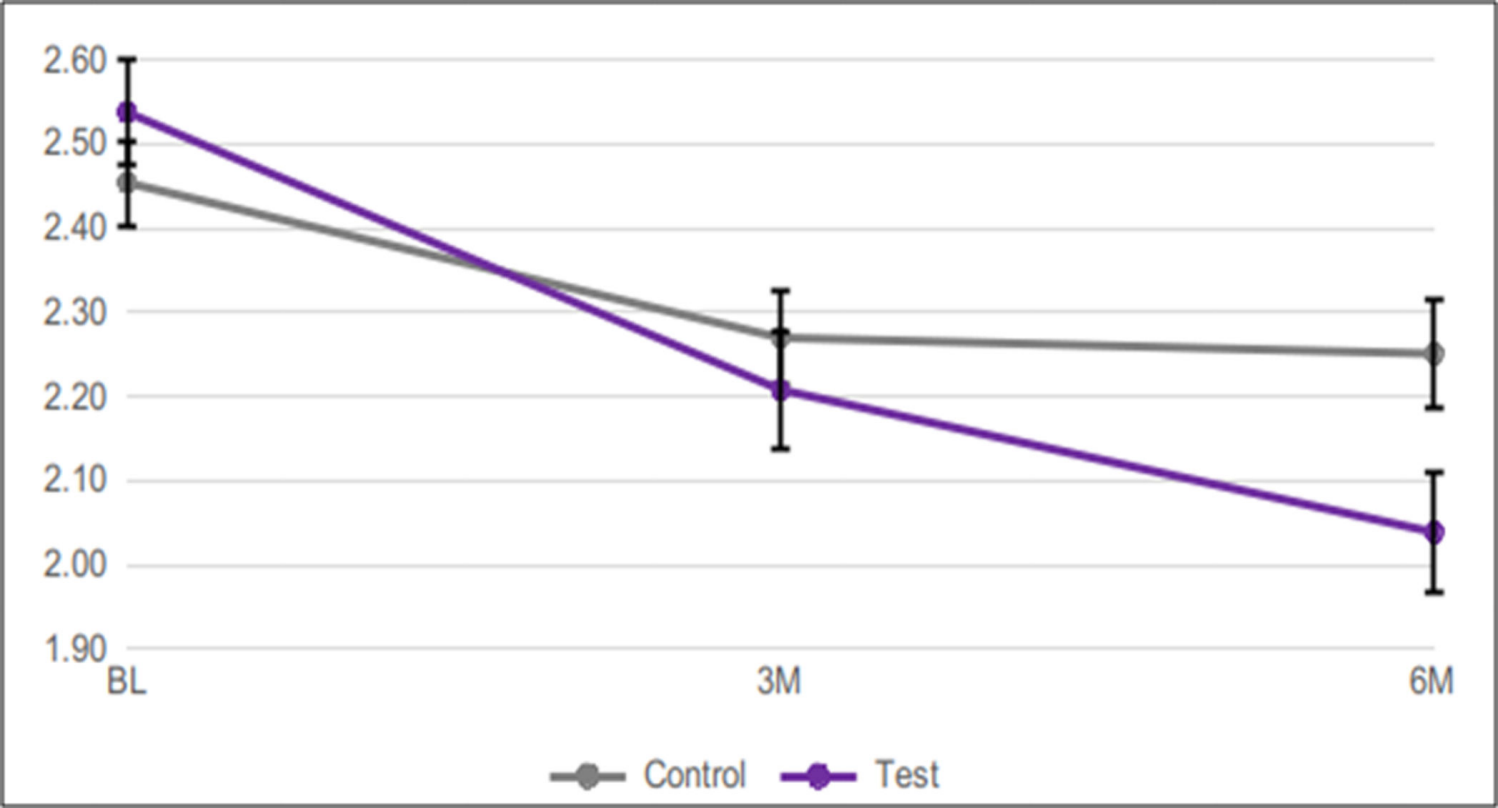
Mean mSBI over 6 months using test*vs.* control dentifrice. Error bars show standard error of mean (SEM).

Mean Baseline mSBI measured 2.55 for the test group, and 2.44 for the control group.After 3 months, mSBI was significantly lower in the test (2.24) *vs.* the control (2.32) group (p=0.0032). This trend continued at the 6-month time point, when mSBI measured 2.10 in the test group *vs.* 2.27 in the control group (p=0.0004)(Table 1 and Figure 3).

### E. Periodontal Pocket Probing Depths

#### 1) All periodontal pockets measurements

**Figure 4:**
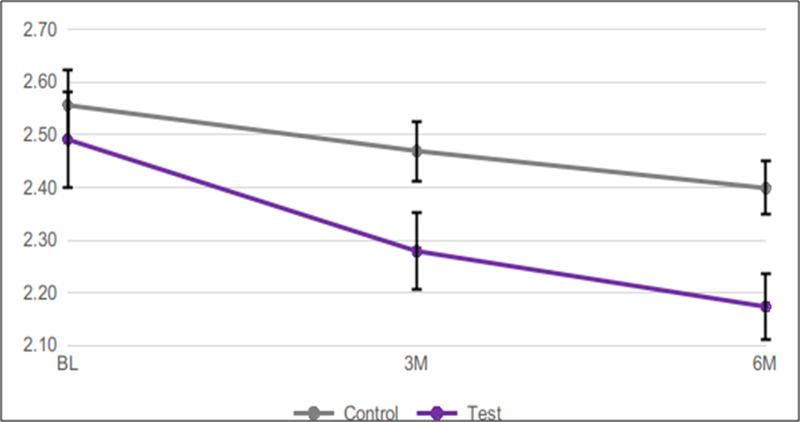
All periodontal pockets: mean probing depths in mm over 6 months using test*vs.* control dentifrice. Error bars show standard error of mean (SEM).

Mean Baseline pocket probing depths measured 2.49 for the test group, and 2.56 for the control group.After 3 months, probing depths were significantly lower in the test (2.28)*vs.* the control (2.47) group (p=0.0140) This trend continued at the 6-month time point, when mean probing depths measured 2.17 in the test group*vs.* 2.40 in the control group (p=0.0108)(Table 1 and Figure 4).

#### 2) Periodontal pockets with baseline measurement >4mm

**Figure 5:**
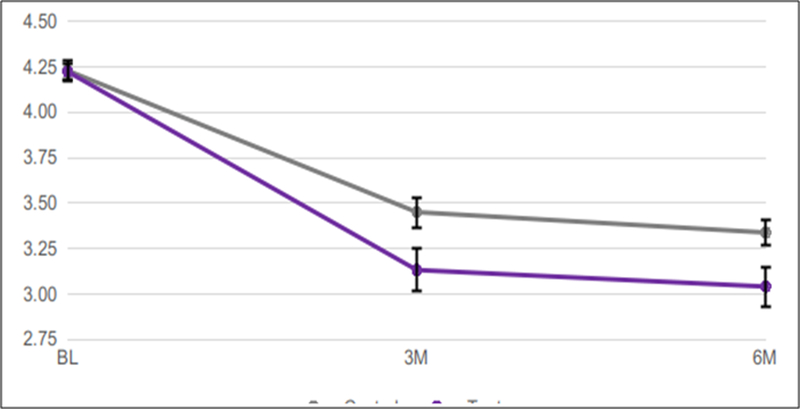
periodontal pockets >4mm: mean probing depths in mm over 6 months using test*vs.* control dentifrice. Error bars show standard error of mean (SEM).

Mean Baseline pocket probing depths measured 4.22 for the test group, and 4.23 for the control group.After 3 months, probing depths were significantly lower in the test (3.13)*vs.* the control (3.45) group (p=0.0142) This trend continued at the 6-month time point, when mean probing depths measured 3.04 in the test group*vs.* 3.34 in the control group (p=0.0142)(Figure 5).

## Discussion

This study is one in a series of projects to evaluate the effects of a novel formulation of dental gel that contains 2.6% edathamil on oral biofilm and periodontal health. Previous studies have demonstrated effective plaque removal and reduced overnight plaque reaccumulation after one-time tooth brushing with the test dental gel[20–24].Additionally, several clinical studies reported that the use of the test gel was associated with lower plaque levels and gingival inflammation as compared to a positive control dentifrice [20–24].In a previous study [21] using a novel in vivo imaging approach, investigators applied multiphoton microscopy and digital imaging techniques to quantify the effects of the test*vs.* control gel on dental plaque. They observed considerably reduced biofilm presence, cohesion and adhesion to the tooth surface after 3 weeks of brushing with the test gel*vs.* a control dentifrice. Moreover, in the subjects using the test gel, the remaining plaque evidenced a fragmented dental biofilm structure, with no apparent changes in the underlying pellicle. Subjects using the control dentifrice also demonstrated reduced plaque levels, but biofilm removal was less extensive with regard to plaque volume, continuity and surface area. Clinical indices were documented in the same study. They paralleled the imaging results in that the clinical indices at the study end-point were also significantly lower in the test group versus the control group.

Previous clinical and imaging studies that demonstrated effective plaque removal and improved gingival health provided the impetus for the current study, raising the question whether the documented short-term effects of the test gel in patients with gingivitis would translate into benefits for patients with mild-to-moderate periodontitis.This study demonstrated that, over a period of 6 months, clinical indices for gingival health as well as periodontal pocket probing depths improved significantly more in subjects using the test gel than in the group using the control gel.

In summary, this study demonstrated that, over a period of 6 months, brushing with test gel twice daily resulted in significantly better gingival health and periodontal probing depths than a control dentifrice. For all variables measured, the differences between test and control dentifrice were significant at 3 months and at 6 months. Further studies are required that include greater patient numbers over longer periods of time.

## Conclusion

A test dental gel more effectively reduced gingival inflammation and bleeding, as well as periodontal pocket probing depths than a control dentifrice. Next steps include more extensive studies with larger numbers of patients and longer study durations in subjects with diverse levels of periodontal health to permit more comprehensive evaluation and understanding of the short- mid- and long-term effects of the test product.

## Figures and Tables

**Table 1:** Summary of Results. Clinical indices and measurements at Baseline, 3 months & 6 months and statistical analysis.*denotes statistically significant at the 0.05 level.

		Control (n=19)	Test (n=14)	Note
**# of Teeth per patient**		26.16	25.07	
**PPD ≥ 4mm**	BL	4.23 mm	4.22 mm	
	3M	3.45 mm (0.78 mm/18.4%)	3.13 mm (1.09 mm/26.0%)	P-value=0.0142*
	6M	3.34 mm (0.89 mm/21.0%)	3.04 mm (1.18 mm/28.1%)	P-value=0.0142*
**All PPD**	BL	2.56 mm	2.49 mm	
	3M	2.47 mm (0.09 mm/3.2%)	2.28 mm (0.21 mm/8.1%)	P-value=0.0140*
	6M	2.40 mm (0.16 mm/5.8%)	2.17 mm (0.32 mm/12.1%)	P-value=0.0108*
**Mean GI Score**	BL	2.45	2.54	
	3M	2.27 (0.18/7.5%)	2.21 (0.33/13.0%)	P-value=0.0152*
	6M	2.25 (0.20/8.3%)	2.04 (0.50/19.6%)	P-value=0.0002*
**Mean mSBI Score**	BL	2.44	2.55	
	3M	2.32 (0.11/4.5%)	2.24 (0.31/12.0%)	P-value=0.0032*
	6M	2.27 (0.17/6.6%)	2.10 (0.45/17.3%)	P-value=0.0004*
